# Examining the use of telehealth to initiate buprenorphine for opioid use disorder treatment

**DOI:** 10.1093/haschl/qxae137

**Published:** 2024-11-02

**Authors:** Yimin Ge, Matthew D Eisenberg, Emma E McGinty, Jiani Yu, Kayla N Tormohlen

**Affiliations:** Johns Hopkins University Bloomberg School of Public Health, Department of Health Policy and Management, Baltimore, MD 21205, USA; Johns Hopkins University Bloomberg School of Public Health, Department of Health Policy and Management, Baltimore, MD 21205, USA; Weill Cornell Medicine, Department of Population Health Sciences, New York, NY 10022, USA; Weill Cornell Medicine, Department of Population Health Sciences, New York, NY 10022, USA; Weill Cornell Medicine, Department of Population Health Sciences, New York, NY 10022, USA

**Keywords:** substance use and addiction, opioid use disorder, telehealth, health service

## Introduction

Telehealth use for opioid use disorder (OUD) treatment increased during the COVID-19 pandemic due to regulatory changes and expanded reimbursement.^1,2^ Telehealth can facilitate the prescribing of buprenorphine, an underused medication for OUD (MOUD) prescribed by office-based clinicians. Telehealth-initiated buprenorphine for OUD has been shown to reduce opioid overdose,^3^ and the option to initiate via telehealth is supported by leading addiction medicine professional groups.^4^ Pandemic-era rules allowed tele-initiation of buprenorphine for new and established patients with OUD. No studies have comprehensively characterized prescribers’ patterns of telehealth vs in-person initiation of buprenorphine for OUD or examined whether these patterns differ by prescriber specialty or patient insurance.

## Methods

This study used IQVIA prescription and medical claims from January 2018 to December 2022, which capture services delivered by 75% of licensed physicians in the United States and 90% of prescriptions dispensed by US retail pharmacies.^5^ The database includes services paid by all insurers. We included providers in the IQVIA data who continuously practiced from 2018 to 2022, treated at least one patient with OUD (ICD-10 F11) annually in that period, and had at least 5 buprenorphine initiations per year. Buprenorphine initiation was defined as a first prescription in 90 days to a patient with an OUD diagnosis medical claim 7 days before or 3 days after;^6^ telehealth initiations were those with telehealth procedure codes (Supplement 1). We measured the proportion of each provider's buprenorphine initiations delivered via telehealth by year from 2018 to 2022 and examined differences by provider type—primary care physicians, behavioral health physicians, nurse practitioners (NPs)/physician assistants (PAs), and emergency medicine physicians (Supplement 2). We also examined providers’ initiation patterns for patients with different primary insurers, defined as the insurance paying the majority of a patient's total claims each year. We conducted a provider-level analysis stratified by patient insurance type to examine the proportion of a provider's total buprenorphine initiations that were done via telehealth among their patients insured by Medicaid, Medicare, commercial, and affordable care act (ACA) Marketplace plans, respectively. All group differences were tested using Welch's *t*-test.

## Results

**Figure 1.**
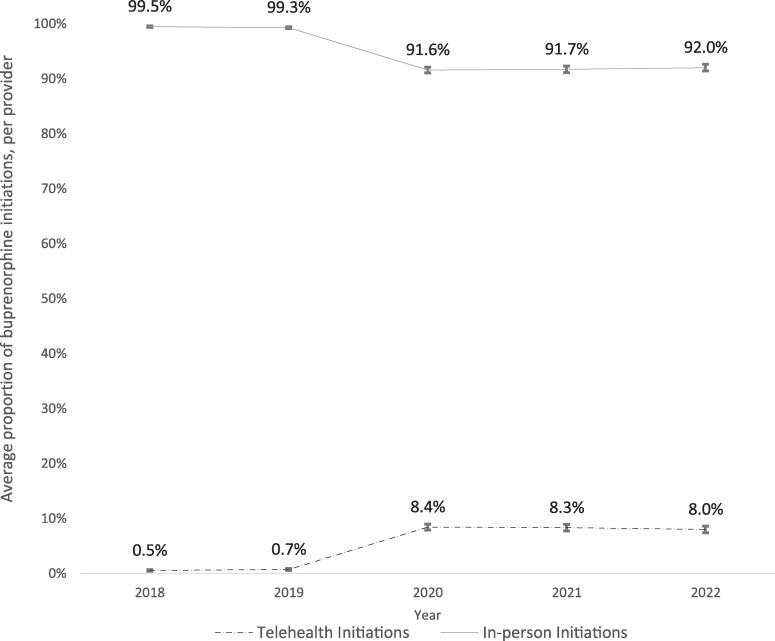
Changes in the initiation of buprenorphine for opioid use disorder treatment via telehealth and in-person care, 2018-2022. Notes: Total number of providers in the sample: n = 3022 in 2018, n = 3366 in 2019, n = 3478 in 2020, n = 3498 in 2021, and n = 3445 in 2022. The average proportion of tele-initiations per provider is 0.5% (95% CI, 0.4%-0.7%) in 2018, 0.7% (95% CI, 0.5%-0.9%) in 2019, 8.4% (95% CI, 7.9%-9.0%) in 2020, 8.3% (95% CI, 7.7%-8.9%) in 2021, and 8.0% (95% CI, 7.4%-8.6%) in 2022. Ninety-five percent CIs are indicated with error bars.

**Figure 2.**
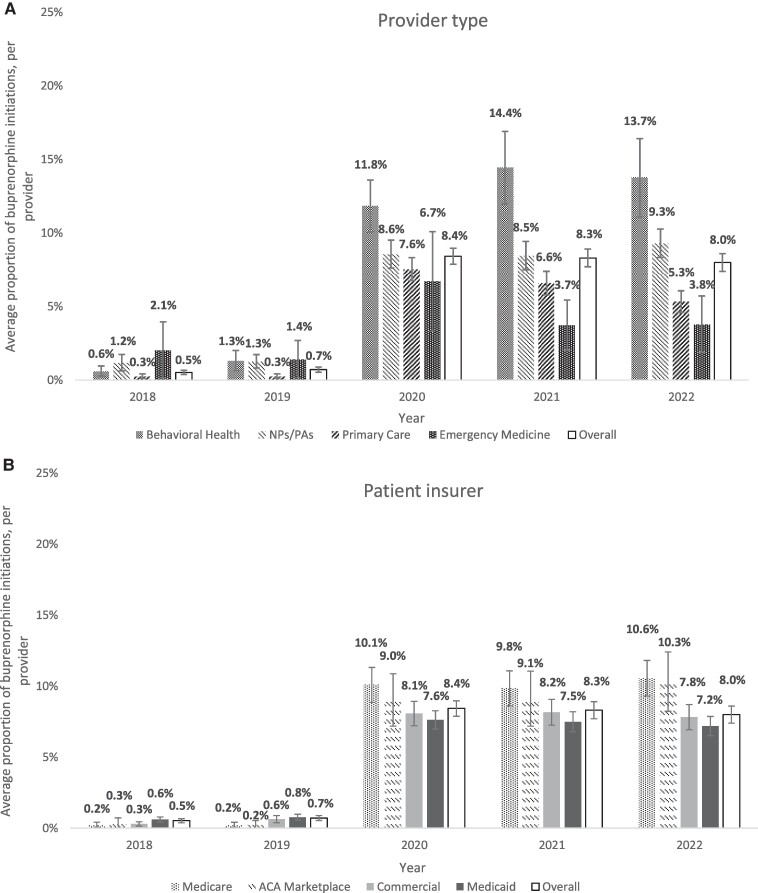
Initiation of buprenorphine for opioid use disorder treatment via telehealth from 2018 to 2022, by provider type and patient insurer. Notes: Total number of providers in the sample: n = 3022 in 2018, n = 3366 in 2019, n = 3478 in 2020, n = 3498 in 2021, and n = 3445 in 2022. Ninety-five percent CIs were indicated with error bars. All differences were significant at the *P* < 0.05 level. Patient insurer types are mutually exclusive.

Our sample included 377 528 unique buprenorphine initiations by 8248 providers for 292 913 patients. The average proportion of buprenorphine tele-initiations for each provider was 0.5% in 2018, 0.7% in 2019, 8.4% in 2020, 8.3% in 2021, and 8.0% in 2022 (Figure 1). The average proportion increased significantly from 2019 to 2020, while the changes from 2020 to 2022 were not statistically significant at the *P* < 0.05 level. Among all provider types, the proportion of buprenorphine initiations via telehealth was <3% in 2018 and 2019, with increases beginning in 2020 (Figure 2A). Behavioral health physicians had the highest average proportion of buprenorphine initiations via telehealth in 2020 and thereafter (11.8% in 2020, 14.4% in 2021, and 13.7% in 2022) statistically significantly higher than all other provider groups each year (Figure 2A). Changes in the proportion of tele-initiations from 2020 to 2022 were not statistically significant for any of the provider types examined. In terms of patient insurance, the proportion of tele-initiations was highest for Medicare patients (10.1% in 2020, 9.8% in 2021, and 10.6% in 2022) and lowest for Medicaid patients (8.4% in 2020, 8.3% in 2021, and 8.0% in 2022), with a statistically significant (*P* < 0.05) difference between Medicare and Medicaid groups, as well as between Medicare and commercial groups in each year (Figure 2B).

## Discussion

Our findings highlight increases in the use of telehealth for buprenorphine initiation beginning in 2020. Building on prior work showing a pandemic-era increase in tele-initiation of MOUD for Medicaid beneficiaries,^1^ our results demonstrate consistent increases in tele-initiation across insured groups. Primary care physicians prescribe most buprenorphine in the United States^7^ but were less likely to initiate buprenorphine via telehealth compared with behavioral health physicians. This may reflect greater comfort with tele-initiation and telehealth capacity among behavioral health physicians.^8^ The increase in NPs/PAs initiating buprenorphine via telehealth may help to overcome access barriers, particularly in rural areas. While these 2 provider groups maintained consistent levels of tele-initiation from 2020 to 2022, suggesting a lasting change in the delivery of OUD treatment as opposed to a temporary response to the pandemic, our findings show declining tele-initiations among primary care and emergency medicine physicians as the pandemic waned. Lower tele-initiation rates among Medicaid patients may indicate unequal access among patient populations or differences in coverage policies, which warrant further study. Our analysis is limited by the infrequent use of telehealth billing codes before 2020, possibly leading to an undercount in 2018-2019. Use of claims data precluded an assessment of the clinical appropriateness of telehealth initiations.

## Supplementary Material

qxae137_Supplementary_Data

## Data Availability

The IQVIA data were accessed through a licensing agreement with Johns Hopkins University. Per the terms of that agreement, we are unable to share the data.
