# Supplementary material for: Examining the use of telehealth to initiate buprenorphine for opioid use disorder treatment
Source: Health Aff Sch. 2024 Nov 2;2(11):qxae137. doi: 10.1093/haschl/qxae137 (PMC11562112; doi:10.1093/haschl/qxae137)
Supplement: qxae137_Supplementary_Data [file qxae137_supplementary_data.zip › HAS_RL_supplement_revision_submission.docx]

Supplement

1. Telehealth Codes and Descriptions

| **Type** | **CPT Code** | **Description** |
| --- | --- | --- |
| Telehealth facility claim | Q3014 | Telehealth facility fee |
| Telehealth facility claim | T1014 | For telehealth transmission, per minute |
| Telehealth, inpatient | G0406 | Follow-up inpatient telehealth, limited, typically 15 min |
| Telehealth, inpatient | G0407 | Follow-up inpatient telehealth, intermediate, typically 25 min |
| Telehealth, inpatient | G0408 | Follow-up inpatient telehealth, complex, typically 35 min |
| Telehealth, inpatient | G0425 | Initial inpatient/ED telehealth, typically 30 mins |
| Telehealth, inpatient | G0426 | Initial inpatient/ED telehealth, typically 50 mins |
| Telehealth, inpatient | G0427 | Initial inpatient/ED telehealth, typically 70+ mins |
| Telehealth, inpatient | G0459 | Inpatient pharmacologic management |
| Telehealth, critical care | 0188T | Remote real-time videoconference critical care |
| Telehealth, critical care | 0189T | Remote real-time videoconference critical care |
| Telehealth, critical care | G0508 | Initial critical care telehealth consult, typically 60 min |
| Telehealth, critical care | G0509 | Subsequent critical care telehealth consult, typically 50 min |
| Telephone | G2025 | RHC/FQHC distant site telehealth service |
| Telephone | 99441 | Non-Face-to-Face Telephone E&M Services, 5-10 mins |
| Telephone | 99442 | Non-Face-to-Face Telephone E&M Services, 11-20 mins |
| Telephone | 99443 | Non-Face-to-Face Telephone E&M Services, 21-30 mins |
| Telephone | 98966 | A nonphysician provider telephone E&M services, 5-10 mins |
| Telephone | 98967 | A nonphysician provider telephone E&M services, 11-20 mins |
| Telephone | 98968 | A nonphysician provider telephone E&M services, 21-30 mins |
| Online E&M | 99421 | Non-Face-to-Face On-Line Digital E&M Service, 5-10 mins |
| Online E&M | 99422 | Non-Face-to-Face On-Line Digital E&M Service, 11-20 mins |
| Online E&M | 99423 | Non-Face-to-Face On-Line Digital E&M Service, 21+ mins |
| Online E&M | G2061 | Online Assessment of established patient by Qualified Nonphysician Healthcare Professional, 5-10 mins |
| Online E&M | G2062 | Online Assessment of established patient by Qualified Nonphysician Healthcare Professional, 11-20 mins |
| Online E&M | G2063 | Online Assessment of established patient by Qualified Nonphysician Healthcare Professional, 21+ mins |
| Online E&M | 98970 | replaced code G2061 in 2021 |
| Online E&M | 98971 | replaced code G2062 in 2021 |
| Online E&M | 98972 | replaced code G2063 in 2021 |
| Online check-in | G2012 | Brief communication technology-based service, e.g. virtual check-in, to an established patient, effective 2021 |
| Online check-in | G2251 | Brief communication technology-based service by a qualified health care professional who cannot report evaluation and management services, not originating from a related E/M service provided within the previous 7 days nor leading to a service or procedure within the next 24 hours or soonest available appointment; 5-10 minutes of medical discussion, effective 2021 |
| Online check-in | G2252 | Brief communication technology-based service by a physician or other qualified health care professional who can report evaluation and management services, not originating from a related E/M service provided within the previous 7 days nor leading to an E/M service or procedure within the next 24 hours or soonest available appointment; 11-20 minutes of medical discussion, effective 2021 |
| **Type** | **CPT Code Modifiers** |  |
| Telephone, modifier | 93 | Modifier for telephone, effective 2022 |
| Telehealth, modifier | GT | Modifier code for synchronous telemedicine |
| Telehealth, modifier | GQ | Modifier code for asynchronous telemedicine |
| Telehealth, modifier | 95 | Modifier code for synchronous telemedicine |
| Telehealth, modifier | G0 | Modifier code for synchronous telemedicine for acute stroke |
| Mental health telehealth, modifier | FR | MENTAL HEALTH - A supervising practitioner was present through a real-time two-way, audio/video communication technology, effective 2023 |
| Mental health telephone, modifier | FQ | MENTAL HEALTH - A telehealth service was furnished using real-time audio-only communication technology, effective 2023 |

1. Analytic Sample

The analytic sample includes providers who practiced continuously each year and treated at least one OUD patient annually. For each provider, the total number of buprenorphine initiations and the proportion via telehealth versus in-person were calculated, with annual averages across all providers. The sample was limited to providers with 5 to 1,000 initiations per year.

In our sample of 8,248 unique providers, the distribution was as follows: 3,142 are primary care physicians, 3,025 are nurse practitioners (NPs) or physician assistants (PAs), 1,162 are behavioral health physicians, and 210 are emergency medicine physicians. The remaining 709 providers were from various other specialties not specified here.

Among the primary care physicians, the majority specialized in family medicine (61.0%), followed by internal medicine (28.2%). The remaining 10.8% were distributed across other specialties. Within the NPs and PAs category, there were 2,431 NPs (80.4%) and 594 PAs (19.6%). Among behavioral health physicians, most of them (80.0%) had a primary or secondary specialty in psychiatry, and nearly half of them (48.9%) had a primary or secondary specialty in addiction medicine. When considering primary specialty alone, most were in psychiatry (65.7%), followed by addiction medicine (16.4%), addiction psychiatry (5.9%), and child & adolescent psychiatry (5.2%). The remaining 6.8% were in geriatric psychiatry, psychiatry neurology, pediatric psychiatry, forensic psychiatry, and psychiatry family medicine. Emergency medicine physicians’ primary specialties included emergency medicine (95.2%), medical toxicology (emergency medicine) (4.3%), and underseas medicine (emergency medicine) (0.5%).

Among patients who received at least one OUD service from providers in our sample, we defined their primary insurer type as the insurer that paid the majority of all a patient’s claims in each year. We then conducted stratified, provider-level analysis among the subset of a provider’s patients with a specific insurance type. In other words, we calculated the proportion of a provider’s total buprenorphine initiations for their Medicaid-insured patients that were done via telehealth, the proportion of a provider’s total buprenorphine initiations for their Medicare-insured patients that were done via telehealth, and so on.
